# Identification of an *NF1* Microdeletion with Optical Genome Mapping

**DOI:** 10.3390/ijms241713580

**Published:** 2023-09-01

**Authors:** Gergely Büki, Anna Bekő, Csaba Bödör, Péter Urbán, Krisztina Németh, Kinga Hadzsiev, György Fekete, Hildegard Kehrer-Sawatzki, Judit Bene

**Affiliations:** 1 Department of Medical Genetics, Clinical Center, Medical School, University of Pécs, 7624 Pécs, Hungary; buki.gergely@pte.hu (G.B.); hadzsiev.kinga@pte.hu (K.H.); 2 HCEMM-SE Molecular Oncohematology Research Group, Department of Pathology and Experimental Cancer Research, Semmelweis University, 1085 Budapest, Hungary; beko.anna@stud.semmelweis.hu (A.B.); bodor.csaba1@semmelweis.hu (C.B.); 3 Bioinformatics Research Group, Genomics and Bioinformatics Core Facility, Szentágothai Research Centre, University of Pécs, 7624 Pécs, Hungary; urban.peter@pte.hu; 4 Pediatric Center, Tűzoltó Street Department, Faculty of Medicine, Semmelweis University, 1094 Budapest, Hungary; nemeth.krisztina@med.semmelweis-univ.hu (K.N.); fekete.gyorgy@med.semmelweis-univ.hu (G.F.); 5 Institut of Human Genetics, University of Ulm, 89081 Ulm, Germany; hildegard.kehrer-sawatzki@uni-ulm.de

**Keywords:** optical genome mapping, OGM, *NF1* microdeletion, structural variation, copy number variation, *NF1* gene

## Abstract

Neurofibromatosis type 1 (NF1) is a clinically heterogeneous neurocutaneous disorder inherited in autosomal dominant manner. Approximately 5–10% of the cases are caused by *NF1* microdeletions involving the *NF1* gene and its flanking regions. Microdeletions, which lead to more severe clinical manifestations, can be subclassified into four different types (type 1, 2, 3 and atypical) according to their size, the genomic location of the breakpoints and the number of genes included within the deletion. Besides the prominent hallmarks of NF1, patients with *NF1* microdeletions frequently exhibit specific additional clinical manifestations like dysmorphic facial features, macrocephaly, overgrowth, global developmental delay, cognitive disability and an increased risk of malignancies. It is important to identify the genes co-deleted with *NF1*, because they are likely to have an effect on the clinical manifestation. Multiplex ligation-dependent probe amplification (MLPA) and microarray analysis are the primary techniques for the investigation of *NF1* microdeletions. However, based on previous research, optical genome mapping (OGM) could also serve as an alternative method to identify copy number variations (CNVs). Here, we present a case with *NF1* microdeletion identified by means of OGM and demonstrate that this novel technology is a suitable tool for the identification and classification of the *NF1* microdeletions.

## 1. Introduction

Neurofibromatosis type 1 (NF1; MIM#162200) is a neurocutaneous disorder caused by loss-of-function mutations in the neurofibromin-1 (*NF1*) gene. It is inherited in an autosomal dominant manner, affecting mainly the peripheral nervous system and the skin [1,2]. NF1 displays complete penetrance and extremely variable expression [3]. It is a tumor predisposition syndrome with an incidence of 1 in 2500–3000 newborns [4]. The most prominent manifestations include café-au-lait spots (CALs), pathognomonic neurofibromas on and/or under the skin, freckling in the axillary/inguinal region and Lisch nodules on the retina [5]. Strong inter- and intrafamilial clinical heterogeneity is observed among patients and certain manifestations are age-dependent [1,2,6,7,8].

The *NF1* gene (NM_000267) is localized on the long arm of chromosome 17 (17q11.2) and encodes a Ras-specific GTPase-activating protein called neurofibromin [6]. Among others, it negatively regulates the Ras/MAPK signaling cascade and participates in the regulation of the mTOR pathway activity [9,10]. The mutation rate of the *NF1* gene is extremely high; in nearly 50% of cases the mutations develop de novo [11]. Novel intragenic mutations occur mainly on the paternally derived chromosomes [11].

Microdeletions affecting the *NF1* gene account for approximately 5–10% of cases associated with neurofibromatosis type 1 [12,13]. Four different subtypes (Type 1, 2, 3 and atypical) can be distinguished (Table 1) based on their localization, size and gene content. The first three types include deletions that exhibit well-defined breakpoints. Therefore, the deletion size and the affected genes are clearly determined. The fourth type includes atypical deletions, which occur between heterogeneous, often non-recurrent breakpoints; thus, the deletion size and the affected genes vary between different deletions. Low copy repeat (LCR) regions harbor the recurrent breakpoints for type-1 and type-3 deletions. LCR regions called NF1-REPa and NF1-REPc are involved in the development of maternally inherited germline type-1 deletions, while NF1-REPb and NF1-REPc participate in the formation of type-3 deletions. Type-2 microdeletions are often formed postzygotically during mitosis by aberrant recombination between the *SUZ12* gene and its pseudogene (*SUZ12P1*) [14,15]. Atypical *NF1* deletions either have a postzygotic origin or are inherited as germline deletions [16]. Several previous studies addressed genotype–phenotype differences between patients with intragenic mutations versus those with *NF1* microdeletions [12,17]. *NF1* microdeletions lead to more severe manifestations with an increased risk of malignancies. Besides the characteristic hallmarks of NF1, specific clinical manifestations occur at an increased frequency in patients with *NF1* microdeletions, such as a large numbers of early-onset neurofibromas, dysmorphic facial features, cognitive deficits and an increased risk for the development of malignant peripheral nerve sheath tumors (MPNSTs) [14,17]. The estimated prevalence of *NF1* microdeletion syndrome (MIM# 613675) is around 1 in 60,000 [14].

Various methodologies can be applied to investigate copy number variants. In the case of *NF1* microdeletions, primarily multiplex ligation-dependent probe amplification (MLPA) and microarray analysis are used for their detection (Table 1). However, accurate characterization of *NF1* microdeletions can be challenging using these techniques because of difficulties in precise breakpoint localization.

While MLPA provides a more targeted approach with higher resolution but limited scope, microarray analysis is capable of detecting large CNVs simultaneously throughout the whole genome. However, microarray analysis is limited in terms of the identification of smaller CNVs and it is not able to detect balanced alterations, such as inversions, balanced translocations or other complex forms [14,17,18,19]. Neither MLPA nor microarray analysis are applicable to precisely characterize *NF1* microdeletion breakpoints. Furthermore, both techniques are not suitable for detecting low levels of mosaicism (below 20%).

A new technology, optical genome mapping (OGM), is rapidly being used in clinical genetics laboratories [20]. OGM is a technology for high-resolution reconstruction of the genome with the help of linearized strands of ultra-high molecular weight DNA. It uses longer DNA sequences to analyse large eukaryotic genomes compared to the current second- and third-generation sequencing methods. The average read lengths are in excess of 200 kbp [21,22]. The OGM workflow does not include tissue culture or PCR amplification; therefore, it decreases the possibility of false overrepresentation of specific abnormalities, such as culture artefacts or PCR bias [20]. DNA molecules are enzymatically labelled at a specific sequence (CTTAAG) [20]. OGM is capable of detecting all classes of structural variants (SVs), such as deletions, duplications, inversions, insertions, translocations and chromosomal aneuploidies, as well as complex rearrangements. It also allows the detection of insertions and deletions as small as 500 bp [23], but it is highly dependent on the distribution of the sequence tags. In addition, a previous study demonstrated that OGM can successfully detect the breakpoints of chromosomal translocations [24]. More and more studies demonstrates the use of OGM as a powerful tool to assess genomic complexity and for diagnostic purposes [20,22,25,26].

In our study, the first in the literature, we aim to leverage the optical genome mapping technique to investigate *NF1* microdeletion in a patient with symptoms characteristic for the *NF1* microdeletion syndrome. Our analysis demonstrates that this novel technology is a suitable tool for the identification and classification of *NF1* microdeletions and to narrow down the localization of the breakpoints.

## 2. Results

The clinical presentation of our 13-year old patient suggested *NF1* microdeletion syndrome. The observed manifestations were, however, moderately different from the characteristic type-1 microdeletion patient group. Therefore, the presence of an atypical *NF1* microdeletion in this patient could not be excluded. MLPA analyses demonstrated a heterozygous deletion affecting the entire *NF1* gene and its upstream and downstream regions. In order to identify the type of the microdeletion more accurately, optical genome mapping was applied.

**Figure 1:**
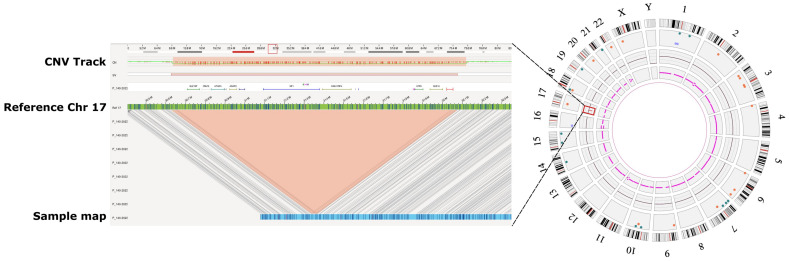
Genome map view and whole genome circos plot. **Right panel**: The whole genome circos plot with a copy number loss visible and highlighted with a red boxed area on the circus plot around the inner circle of the CNV track of chromosome 17. **Left panel**: The genome map view of the interstitial deletion of 17q11.2 region highlighted in the CNV track and the structural variation (SV) track. Green horizontal bar displays the reference genome with the OGM labels marked with blue vertical lines. Blue horizontal line with small vertical lines demonstrates our sample genome map with labels.

The OGM analyses revealed a heterozygous deletion spanning 1,447,330–1,467,226 bp, affecting 14 protein-coding and 5 microRNA genes, which corresponds to the type-1 microdeletion group (Figure 1).

Analysis of the OGM results demonstrated 17 further copy number variations. Among them were 13 deletions and 4 insertions. However, none of the affected genes associated with pathogenicity and, hence, these CNVs are unlikely to contribute to the clinical phenotype of our patient.

We investigated the ability of OGM in *NF1* microdeletion classification and the resolution capability of this technique. For this purpose we compared the genomic position of probes flanking the deletion breakpoint regions included in OGM and the SALSA MLPA kit P122-D1 NF1 kit (Table 2). The genomic position of these probes is indicated according to GRCh37.

According to the MLPA analysis, the region which includes the centromeric deletion breakpoint spans 268,971 bp and the telomeric breakpoint region encompasses 345,179 bp. By contrast, the centromeric deletion breakpoint region, as determined by OGM, encompasses only 8.736 bp and the telomeric deletion breakpoint region spans 11,160 bp. If the predicted size of the deletion is considered, it would range between 1,290,184 and 1,904,334 bp according to MLPA. However, based on the OGM results, the deletion size is between 1,447,330 and 1,467,226 bp. Consequently, OGM provides a much higher resolution of the deletion breakpoints than MLPA and a much more precise prediction of the deletion size. Furthermore, the deletion breakpoint regions, as determined by OGM, harbor the PRS1 and PRS2 breakpoint hotspots for type-1 *NF1* microdeletions, as previously determined by breakpoint-spanning PCRs [27]. These breakpoint-spanning PCRs, performed in order to identify type-1 *NF1* deletion breakpoints, are laborious and not used during routine diagnostic procedures. Our results suggest that OGM represents a powerful technique to predict type-1 *NF1* deletions with breakpoints in the PRS1 and PRS2 hotspots, without the application of cumbersome breakpoint-spanning PCRs.

We compared the phenotype of our proband with the phenotype of six of our previously published pediatric patients with type-1 *NF1* microdeletion, in whom the breakpoint analyses were performed with microarray analysis [17]. Interestingly, while our 13-year-old patient did not manifest coarse face, macrocephaly and overgrowth, these symptoms were frequent among our previously published cases (five in six). (Supplementary Table S1) Comparing the size of the detected deletions revealed that our 13-year-old patient (#140) harbors the largest deletion (Supplementary Figure S1). Furthermore, this deletion was found to be larger than those deletions characterized by Summerer et al. [27] in a large patient cohort (n = 236) with type-1 *NF1* microdeletion.

We performed an in silico analysis using the UCSC genome browser, in order to search for potential regulatory elements that might be associated with the differences in clinical manifestations of our proband and our previously published pediatric patients with type-1 *NF1* microdeletion. A number of transcription factor binding sites (TFBSs) have been found (Supplementary Table S2); however, based on our current knowledge none of them has an effect on the phenotype of our patients.

## 3. Discussion

*NF1* microdeletion syndrome, which accounts for 5–10% of all NF1 patients, is characterized by specific manifestations. The most common are the type-1 deletions and, thus, the majority of our knowledge about the clinical phenotype associated with this syndrome comes from this group of patients. According to previous observations, macrocephaly, tall stature, skeletal abnormalities, increased number and frequency of neurofibromas, developmental delay and/or intellectual disability and dysmorphic features, including large hands and feet, coarse facial features, and facial dysmorphism, occur more frequently in patients with type-1 deletions as compared to patients with pathogenic variants within the *NF1* gene (Table 3). In addition, an increased risk and more intense progression of MPNSTs are more likely to occur in patients with type-1 microdeletion [17].

Our patient presented not all characteristic features of *NF1* microdeletion syndrome (Table 3). Therefore, the existence of an atypical *NF1* microdeletion could not be excluded. Macrocephaly, tall stature, large hands and feet as well as coarse facial features, which are often observed in patients with type-1 microdeletions, were absent in our patient. However, our patient was only 13 years old and, hence, the full phenotype associated with *NF1* microdeletion syndrome may not yet be observed. Importantly, OGM examination identified a type-1 microdeletion in our patient. Consequently, OGM presents a powerful technique to identify and classify type-1 *NF1* deletions in particular in patients who do not exhibit all clinical features frequently associated with *NF1* microdeletion syndrome or in patients that are quite young and may not, as yet, clearly exhibit the full phenotypic spectrum of the disease manifestations.

Type-1 deletion harbors 14 protein coding genes and 5 microRNA genes. It has been assumed that some of the genes co-deleted with *NF1* have an effect on the clinical manifestation observed in NF1 deletion patients, thus affecting the severity of the disease [14]. Haploinsufficiency of certain genes may contribute to dysmorphic facial features, overgrowth and reduced cognitive capability or heart defects, or might have a tumor suppressive function. Childhood overgrowth is often exhibited in patients with large *NF1* deletions [30]. Two plausible genes has been linked to this manifestation: the *SUZ12* and the *RNF135* genes [31,32,33]. Although the majority of our previously characterized patients presented this feature, the patient tested by OGM did not manifest it; however, her deletion comprised these two genes. Patients with type-1 deletion harbor almost the same deletion; nevertheless, they demonstrate notable clinical variability. This may be related to the unique genomic architecture of the patients.

Large genomic deletions may affect not only protein coding genes, but various regulatory elements as well. There is growing evidence that gene regulatory elements are not only involved in the pathogenesis of common and complex diseases, but may also contribute to the development of Mendelian diseases as well [34]. Structural variants (SVs) potentially separate regulatory elements from their target gene, thereby SVs can indirectly influence the expression level of a gene by altering the spatial relationship between a regulatory element and a gene [35]. In this study, we performed an in silico investigation of the genomic environment surrounding the breakpoints detected in our proband and our previously published pediatric patients with type-1 deletion. Although we have found several TFBSs, based on our current knowledge, they are unlikely to have an impact on the clinical symptoms of our patients and, thus, do not explain the observed difference in the clinical manifestations among our patients.

**Figure 2:**
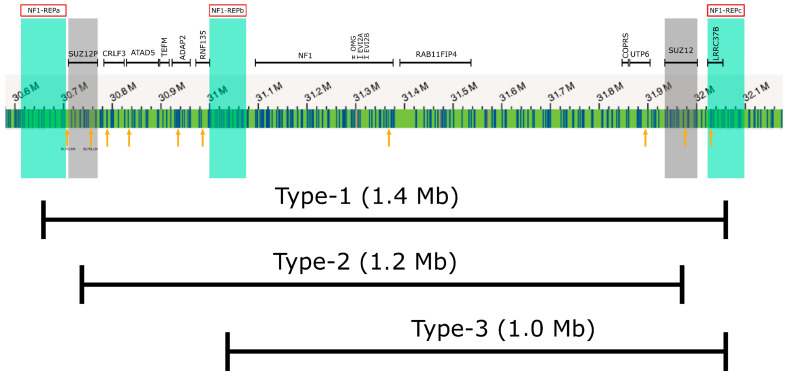
Probe localization on the 17q11.2 microdeletion region. The affected genes and NF1-REP regions in red boxes are displayed schematically at the top of the figure. Vertical blue lines on the green bar show the OGM labels. Orange arrows depicts the NF1 area MLPA probe positions. OGM probes are capable of differentiating between the different types of *NF1* microdeletions.

One possible laboratory technique for the investigation of *NF1* microdeletions is the NF1 area MLPA assay. However, because of the distribution and localization of the corresponding MLPA probes, the accurate characterization of the different *NF1* microdeletion types is limited. In order to differentiate between the types of microdeletions more accurately, a higher probe density is required. OGM, the state-of-the-art technology, includes a lot of probes/markers located in the *NF1* microdeletion region. Therefore, it represents a promising tool for the classification of the various *NF1* microdeletion types. Figure 2 demonstrates that OGM has a higher resolution compared to the NF1 area MLPA assay; therefore, OGM is better suited to classify the various microdeletions more accurately than MLPA.

In addition to MLPA, other techniques for CNV detection are available. A previous study [36] compared specific characteristics of CNV calls with a SNP array, and short- and long-read technologies to highlight their most important features. According to their findings, long reads provide better CNV calls in regions which are not easy to access by short reads or arrays due to repeats and problems with mappability. Mapping and analyzing the numerous highly similar, often repetitive, regions in the human genome has proven to be a challenging task using short-read technologies. However, repetitive sequences, such as LCR regions, often contribute to CNV formation; therefore, the mapping of these regions could be crucial and beneficial [20,36].

OGM uses long molecules, which helps to detect regions that are challenging to map. The specific sequences used in OGM are located approximately every 5000 base pairs; however, the specific sequence tags are not evenly distributed throughout the genome. This may potentially impact the resolution of certain genomic regions [20]. In terms of its resolution, OGM is comparable to aCGH. However, OGM is capable of detecting balanced CNVs, which have been shown to contribute to congenital anomalies by disrupting genes, and potentially affecting regulatory interactions [37,38]. A limitation of OGM is that Robertsonian and other whole-arm translocations with breakpoints located in centromeric regions are not detectable by OGM [22].

In addition to MLPA and microarray analysis, OGM is also an applicable method for the diagnosis of *NF1* microdeletions. Application of other technologies in addition to MLPA can be useful to characterize the localization of the deletion breakpoints more accurately. This may be particularly relevant in patients who present with atypical manifestations not observed in the majority of patients with type-1 *NF1* deletions. The presence of non-characteristic clinical symptoms in patients with an *NF1* microdeletion might suggest the existence of other additional pathogenic variants. OGM provides information throughout the whole genome, which can be very helpful in terms identifying such additional pathogenetically relevant CNVs.

In diagnostic laboratories, OGM technology can potentially replace the standard diagnostic testing strategy based on simultaneous or sequential use of multiple technologies such as karyotyping, fluorescence in situ hybridization (FISH), MLPA and chromosomal microarray (CMA), since it is capable of detecting the full spectrum of structural variations (SVs) in a single assay with a rapid turn-around time. The limitation of OMG, however, is that it is not capable of analyzing previously collected samples stored in DNA biobanks, since it requires intact, large, non-fragmented DNA molecules for the analysis. Because of its high resolution, OGM is capable of characterizing breakpoints more precisely and cost-effectively, by replacing the time-consuming and laborious PCR and sequencing-based technologies. In addition, the simplicity in data interpretation substantially reduces the need for complex and/or customized bioinformatic pipelines and, thus, the burden of trained experts as well. These advantages considerably reduce the overall cost of the diagnostic workflow. Moreover, several publications have demonstrated OGM’s ability to identify novel, clinically relevant genomic abnormalities, to detect balanced SVs and to localize additional materials; moreover, besides CNV analysis, it is capable of detecting repeat expansions [22,25,39] as well, thereby increasing diagnostic yield in genetic patient care.

## 4. Materials and Methods

### 4.1. Patient’s Clinical Presentation

A peripheral blood sample of a patient with suspected neurofibromatosis type 1 was sent to our laboratory for testing of the *NF1* gene. At the age of two years, the female patient demonstrated characteristic manifestations, such as CALs, axillary freckling, skeletal anomaly (mild form of pectus excavatum) and T2 hyperintensities. Cranial MRI examination was carried out at the age of four and the results were consistent with the suspected neurofibromatosis, mainly with cerebellar foci and a very low degree of left optic nerve involvement. The follow-up examination at the age of 13 revealed multiple subcutaneous neurofibromas. She presented facial dysmorphic features including hypertelorismus, low-set ears, a broad nasal bridge, and micro- and retrognathia. Her height and weight were under the age-appropriate value. The patient exhibited speech and learning difficulties (dyscalculia) and visual disturbances (hypermetropia). Her skeletal anomaly was complemented with bilateral second and third finger syndactyly. Cranial MRI examination at the age of 13 did not reveal malignant transformation. The patient fulfilled the diagnostic NIH criteria for NF1. Her clinical phenotype suggested the presence of an *NF1* microdeletion, even though some of the features frequently observed in patients with *NF1* microdeletions including macrocephaly, tall stature, large hands and feet, as well as coarse facial features, were absent in our patient (Table 3).

The study was approved by the ethics committee of the University of Pecs (Protocol 8581-7/2017/EUIG). Written informed consent was obtained from the patient or their legal guardians and peripheral blood samples were collected. All experiments were performed in accordance with the Helsinki Declaration of 1975 and with the Hungarian legal requirements of genetic examination, research and biobanking.

### 4.2. Sample Preparation and MLPA Analysis

DNA was isolated from peripheral blood leukocytes with an E.Z.N.A.^®^ Blood DNA Maxi kit (Omega BIO-TEK, Norcross, GA, USA). A NanoDrop 2000 spectrophotometer (Thermo Fisher Scientific, Waltham, MA, USA) was used to measure the concentration and purity of extracted DNA.

To screen for large deletions and duplications of the *NF1* gene, MLPA analysis was performed using the P081-D1 and P082-C2 SALSA MLPA kits (MRC-Holland, Amsterdam, The Netherlands). The two probe mixes contained together one probe for each exon, three probes for exon 1, one probe for intron 1 and two probes for the exons 15, 21, 23, 51 and 58 of the *NF1* gene. In addition to probes along the entire length of the *NF1* gene, the kit also include an upstream and a downstream probe. According to the manufacturer’s instructions, 100–200 ng of genomic DNA from the patient and the same amount of three control genomic DNA samples were used for hybridization. Amplification products were separated by capillary electrophoresis on an ABI 3130 Genetic Analyzer (Life Technologies, Carlsbad, CA, USA) and the results were analyzed using Coffalyser software (MRC-Holland, Amsterdam, The Netherlands, v.220513.1739). Each MLPA signal was normalized and compared to the corresponding peak area obtained from the three control samples. Deletions and duplications of the targeted regions were suspected when the signal ratio exceeded 30% deviation.

### 4.3. Optical Genome Mapping

During the sample preparation, ultra-high molecular weight (UHMW) genomic DNA (gDNA) was extracted from 1.5 million cells of the frozen blood sample using the SP Blood and Cell Culture DNA Isolation Kit (Bionano, San Diego, CA, USA), following the manufacturer’s SP Blood and Cell Culture DNA Isolation Protocol. After the overnight homogenization and concentration, and quantification using the Qubit™ dsDNA BR Assay Kit (Thermo Fisher Scientific, Waltham, MA, USA), 750 ng of highly viscous UHMW gDNA was fluorescently labeled using the Bionano Prep Direct Label and Stain (DLS) Kit (Bionano, San Diego, CA, USA). Following the concentration quantification (Qubit™ dsDNA HS Assay Kit, Thermo Fisher Scientific, Waltham, MA, USA), 19.5 uL labeled gDNA was loaded into the flowcell of the Saphyr Chip G2.2 (Bionano, San Diego, CA, USA). In the channel-system of the chip, the labeled gDNA molecules became linearized while floating into the nanochannels, enabling capture of the fully stretched molecules by the optical system. To reach the 150X effective coverage, 500 Gbp data was collected from the sample using the Bionano Saphyr System (Bionano, San Diego, CA, USA) [40]. The analysis of the data was performed using the De Novo Assembly Annotation of the Bionano Access software (bionano.com/software-downloads/). After filtering out the structural variants that were present in more than 1% of the Bionano’s human control SV sample database [41], the masking of high-variance regions was performed using the sv_mask.bed file of the Bionano Access software, to filter out the variants in the areas around the centromeric and telomeric regions, where the alignment of the molecules could be unreliable [23]. The remaining SVs were considered as true findings.

### 4.4. Analyses of Regulatory Elements within the NF1 Microdeletion Region

In silico analysis was performed in order to determine regulatory elements within the *NF1* microdeletion region, focusing on the surrounding area of the breakpoints corresponding to type-1 *NF1* microdeletions. The breakpoints were converted into the hg19 genome build, where it was necessary. For this purpose, the OReganno identifiers were collected from the UCSC database. Additional tracks were ENCODE Transcription Binding Factors, base positions and UCSC genes, according to the GRCh37/hg19 assembly. All ORegAnno identifiers with the associated transcription factor names and their genomic positions were collected between the known deleted OGM breakpoints of our case and the known deleted aCGH breakpoints of previous cases.

The Open Regulatory Annotation (ORegAnno) demonstrates literature-curated, experimentally proven regulatory regions, polymorphisms and transcription factor binding sites (TFBSs). The data associated with the regulatory elements are from the PAZAR and JASPAR datasets. The PAZAR database serves as a publicly accessible repository for annotations of regulatory sequences and transcription factors. The JASPAR database encompasses non-redundant and curated, experimentally determined TFBSs in different eukaryote organisms.

## 5. Conclusions

*NF1* microdeletion syndrome is frequently associated with more severe clinical manifestations as observed in the general NF1 patient cohort. Specific clinical features include dysmorphic facial features, macrocephaly, large hands and feet, overgrowth, global developmental delay, cognitive disability and increased risk of malignancies. An early diagnosis, specialized long-term clinical care and closer follow-up of patients with *NF1* microdeletions are essential. Genes co-deleted in addition to the *NF1* gene can have a significant effect on the clinical manifestations; therefore, identification of all of the genes included in the deletion is important. Besides MLPA and microarray analysis, optical genome mapping could serve as an important method to identify and characterize *NF1* microdeletions. Here, we present the case of a 13-year-old girl with an early onset of multiple subcutaneous neurofibromas, learning disabilities and facial dysmorphic features suggestive of *NF1* microdeletion syndrome, even though some of the features frequently associated with this syndrome were absent, including macrocephaly, tall stature and large hands and feet, as well as coarse facial features. Nevertheless, OGM analysis revealed a type-1 *NF1* microdeletion in this patient. Our findings demonstrate that this novel technology is a powerful tool for the identification and classification of *NF1* microdeletions, which is particularly important in patients who do not exhibit all of the clinical features frequently associated with *NF1* microdeletion syndrome and it helps to narrow down the localization of the breakpoints.

## Figures and Tables

**Table 1:** Different types of *NF1* microdeletions with their frequencies and the molecular genetic methods suitable for their classification.

Microdeletion Type	Type-1	Type 2	Type 3	Atypical
Frequency	70–80%	~10%	1–4%	10–20%
Suitable method for appropriate deletion classification	MLPA Probemix P122-D2 NF1-area or microarray analysis	Microarray analysis	Microarray analysis	Microarray analysis
Affected genes	14 protein coding and 5 miRNA	13 protein coding and 5 miRNA	9 protein coding and 5 miRNA	Heterogeneous

**Table 2:** Genomic positions of the MLPA and OGM probes flanking the deletion breakpoint regions.

MLPA probe positions (NF1 Area)			
Preceding probe not included in the deletion	Centromeric probe deleted	Telomeric probe deleted	Following probe not deleted
CPD (28,789,435)	SUZ12P (29,058,406)	LRRC37B (30,348,590)	ZNF207 (30,693,769)
OGM probe positions			
Preceding probe not included in the deletion	Centromeric probe deleted	Telomeric probe deleted	Following probe not deleted
28,946,383	28,955,119	30,402,449	30,413,609

**Table 3:** The occurrence of the hallmark manifestations of *NF1* microdeletion syndrome in the different patient groups.

Distinctive Characteristic Symptoms		Patients with Intrageneic Pathogenic *NF1* Variants [28,29]	*NF1* Microdeletion Patients	Type 1 Microdeletion [14,17,28]	Type 2 (non-Mosaic) Microdeletion [17,28]	Atypical Microdeletion [17]	Our Patient (#140)
Dysmorphic features	Facial dysmorphism	Rare	Frequent	67%	60%	16%	X
	Coarse face	Absent	Frequent	67%	N/A	26%	-
	Large hands, feet	Absent	Frequent	67%	60%	16%	-
Neurofibromas	Cutaneous	91%	More, earlier	86%	60% *	42%	-
	Subcutaneous	58%	More, earlier	76%	60% *	16%	X
	Plexiform	43–50%	More, earlier	76%	60% *	21%	-
Macrocephaly		33.9%	More frequent	58%	60%	16%	-
Tall stature		Not typical	Frequent	58%	0%	N/A	-
Skeletal anomalies including scoliosis		43%	Frequent	92%	80%	58%	X
DD/ID		45%	Frequent	75%	N/A	47%	-
Learning difficulties		30–60%	Frequent	75%	80%	11%	X

*—limited sample size, DD—developmental delay, ID—intellectual disability, N/A—not assessed or no data available, - absent, X—present.

## Data Availability

The data presented in this study are available on request from the corresponding author.

## Supplementary Materials

The following supporting information can be downloaded at: https://www.mdpi.com/article/10.3390/ijms241713580/s1.
