# Supplementary material for: Identification of an NF1 Microdeletion with Optical Genome Mapping
Source: Int J Mol Sci. 2023 Sep 1;24(17):13580. doi: 10.3390/ijms241713580 (PMC10487413; doi:10.3390/ijms241713580)
Supplement: Supplementary file 1 [file ijms-24-13580-s001.zip › Supplementary Figure S1.pdf]

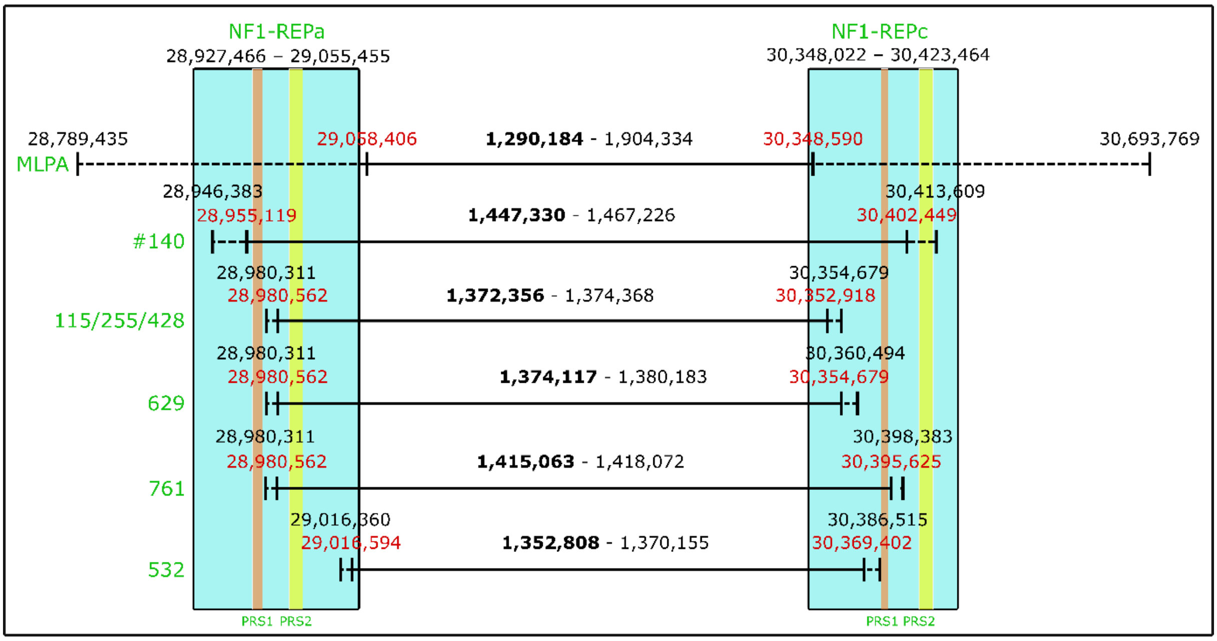

Supplementary Figure S1. Schematic representation of the detected deletions in our patient demonstrated in this article (#140) and in our previously published cases. Solid lines indicate the deleted regions, dotted lines indicate the possibly deleted regions. The size of the known deletion is indicated in bold, while the size of the potentially deleted region is indicated with default font. The genomic positions of the breakpoints are indicated according to GRCh37. Red colour represents the breakpoints involved in the deletion, black colour indicates the following distal or proximal probe position that was not affected by the deletion. Light blue vertical rectangle shows the NF1-REPa and NF1-REPc regions. PRS1 is displayed by brown vertical rectangle, and PRS2 is represented by yellow vertical rectangle.
