# Supplementary material for: Identification of an NF1 Microdeletion with Optical Genome Mapping
Source: Int J Mol Sci. 2023 Sep 1;24(17):13580. doi: 10.3390/ijms241713580 (PMC10487413; doi:10.3390/ijms241713580)
Supplement: Supplementary file 1 [file ijms-24-13580-s001.zip › Supplementary Table S1.pdf]

Supplementary Table S1. Clinical features observed in pediatric patients with type-1 *NF1* microdeletions

	**Applied method**	OGM	CMA					
	**Patients**	# 140	115/ NF	255/ NF	428/ NF	532/ NF	629/ NF	761/ NF
	**Gender**	F	F	M	M	M	F	M
	**Age of onset**	at birth	5 mo	at birth	at birth	12 y	at birth	at birth
	**Age at examination**	13 y	9 y	14 y	5 y	14 y	4.5y	9 y
**Dysmorphic features**	Facial dysmorphism	X	X	X	X	X	X	-
	Hypertelorism	X	X	X	X	X	X	-
	Facial asymmetry	-	-	-	-	X	-	X
	Coarse face	-	-	X	X	X	X	X
	Broad neck	-	-	X	-	-	-	-
	Large hands, feet	-	X	X	X	X	X	X
**Skin manifestations**	CALs	X	X	X	X	X	X	X
	Freckling	X	X	X	X	-	X	X
	Excess soft tissue	-	-	X	X	-	X	-
	SBC neurofibromas	X	X	X	X	-	-	X
	CT neurofibromas	-	-	-	-	-	-	-
	PL neurofibromas*	-	-	-	-	-	-	-
**Education and behavior problems**	SDiCD	-	-	X	X	X	X	X
	Learning difficulties	X	-	X	-	X	X	X
	Speech difficulties	X	-	X	X	X	X	X
	IQ < 70	-	-	-	-	-	-	-
	ADHD	-	-	-	X	-	-	-
**Skeletal manifestations**	Skeletal anomalies	X	X	X	X	X	X	X
	Scoliosis	-	-	X	-	X	-	-
	Pectus excavatum	X	X	-	X	X	-	-
	Bone cysts	-	n.d.	-	n.d.	-	-	-
	Joint hyperflexibility	-	-	-	X	-	-	-
	Macrocephaly	-	X	X	X	-	X	X
**Neurological manifestations**	Muscular hypotonia	-	-	X	-	-	-	X
	Headache	-	-	-	X	-	-	-
	Coordination problem	-	-	X	X	-	-	X
	MPNST	-	-	-	-	-	-	-
	Spinal neurofibromas	-	n.d.	n.d.	n.d.	-	n.d.	X
	T2 hyperintensities	X	X	X	X	-	X	X
**Ocular manifestations**	Visual disturbance	X	-	-	-	X	-	-
	Lisch nodules	-	-	X	-	-	-	-
	Strabismus	-	-	-	-	X	-	-
	Optic gliomas	-	-	-	-	-	X	-
**Development. problems**	Tall stature	-	X	X	-	X	X	X

OGM, optical genome mapping; CMA, chromosomal microarray; CALs, café-au-lait spots; CT/SBC/PL, cutaneous/subcutaneous/plexiform neurofibroma; SDiCD, significant delay in cognitive development; ADHD, Attention deficit hyperactivity disorder; MPNST, malignant peripheral nerve sheath tumours;
*means externally observable plexiform neurofibroma
-, absent; X, present, n.d., not determined

Patients 115, 255, 428, 532, 629, 761 were reported by Büki et al. [1]

1. Buki, G.; Zsigmond, A.; Czako, M.; Szalai, R.; Antal, G.; Farkas, V.; Fekete, G.; Nagy, D.; Szell, M.; Tihanyi, M.; et al. Genotype-Phenotype Associations in Patients With Type-1, Type-2, and Atypical NF1 Microdeletions. *Front Genet* **2021**, *12*, 673025, doi:10.3389/fgene.2021.673025.
