# Supplementary material for: Identification of an NF1 Microdeletion with Optical Genome Mapping
Source: Int J Mol Sci. 2023 Sep 1;24(17):13580. doi: 10.3390/ijms241713580 (PMC10487413; doi:10.3390/ijms241713580)
Supplement: Supplementary file 1 [file ijms-24-13580-s001.zip › Supplementary Table S2.pdf]

Supplementary Table S2. Transcription factor binding sites (TFBSs) affected in our current patient (#140) compared to the previously demonstrated pediatric patients with type-1 *NF1* microdeletions

		Affected TFBS	
**Patients**	**Breakpoints***	**Left side**	**Right side**
**#140**	28,955,119-30,402,449	-	-
**115/NF**	28,980,562-30,352,918	USF1 (3x)	CEBPA, USF1 (x2), CTCF, CEBPB, BATF::JUN
**255NF**	28,980,562-30,352,918	USF1 (3x)	CEBPA, USF1 (x2), CTCF, CEBPB, BATF::JUN
**428NF**	28,980,562-30,352,918	USF1 (3x)	CEBPA, USF1 (x2), CTCF, CEBPB, BATF::JUN
**532/NF**	29,016,594-30,369,402	USF1 (3x), BATF::JUN, YY1, FOXP1, ZNF263 (2x), CEBPB, PRDM14	CEBPA, USF1 (2x), CTCF, CEBPB, BATF::JUN
**629/NF**	28,980,562-30,354,679	USF1 (3x)	CEBPA, USF1 (2x), CTCF, CEBPB, BATF::JUN
**761/NF**	28,980,562-30,395,625	USF1 (3x)	CEBPB, BATF::JUN

* The breakpoints were converted into GRCh37/hg19 genome build
-, absent

Those TFBSs were collected which were affected in the current deletion (#140) compared to the previously demonstrated pediatric patients. In order to do this, we compared the known deleted position of our current case (OGM) and the other patients (aCGH) with the help of UCSC genome browser, both left and right side. Patients 115, 255, 428, 532, 629, 761 were reported by Büki et al. [1]

1. Buki, G.; Zsigmond, A.; Czako, M.; Szalai, R.; Antal, G.; Farkas, V.; Fekete, G.; Nagy, D.; Szell, M.; Tihanyi, M.; et al. Genotype-Phenotype Associations in Patients With Type-1, Type-2, and Atypical NF1 Microdeletions. *Front Genet* **2021**, *12*, 673025, doi:10.3389/fgene.2021.673025.
